# Fine-Grain Segmentation of the Intervertebral Discs from MR Spine Images Using Deep Convolutional Neural Networks: BSU-Net

**DOI:** 10.3390/app8091656

**Published:** 2018-09-14

**Authors:** Sewon Kim, Won C. Bae, Koichi Masuda, Christine B. Chung, Dosik Hwang

**Affiliations:** 1 School of Electrical Engineering, Yonsei University, Seoul 03722, Korea; sewon.kim@yonsei.ac.kr; 2 Department of Radiology, VA San Diego Healthcare System, San Diego, CA 92161-0114, USA; wbae@ucsd.edu (W.C.B.); cbchung@ucsd.edu (C.B.C.); 3 Department of Radiology, University of California-San Diego, La Jolla, CA 92093-0997, USA; 4 Department of Orthopedic Surgery, University of California-San Diego, La Jolla, CA 92037, USA; koichimasuda@ucsd.edu

**Keywords:** intervertebral disc, segmentation, convolutional neural network, fine grain segmentation, U-net, deep learning, magnetic resonance image, lumbar spine

## Abstract

We propose a new deep learning network capable of successfully segmenting intervertebral discs and their complex boundaries from magnetic resonance (MR) spine images. The existing U-network (U-net) is known to perform well in various segmentation tasks in medical images; however, its performance with respect to details of segmentation such as boundaries is limited by the structural limitations of a max-pooling layer that plays a key role in feature extraction process in the U-net. We designed a modified convolutional and pooling layer scheme and applied a cascaded learning method to overcome these structural limitations of the max-pooling layer of a conventional U-net. The proposed network achieved 3% higher Dice similarity coefficient (DSC) than conventional U-net for intervertebral disc segmentation (89.44% vs. 86.44%, respectively; *p* < 0.001). For intervertebral disc boundary segmentation, the proposed network achieved 10.46% higher DSC than conventional U-net (54.62% vs. 44.16%, respectively; *p* < 0.001).

## 1. Introduction

Low back pain is a common disease in modern society. It can be caused by disorders of lumbar components such as an intervertebral disc, paraspinal muscle, and vertebral body. Therefore, it is important to examine the specific components of the lumbar spine for accurate diagnosis and treatment. Assessment of the intervertebral disc is particularly important since its shape is liable to physiological (age-related) and pathological changes [1,2]. Magnetic resonance (MR) imaging is a very effective non-invasive imaging modality for obtaining such information. However, segmentation of intervertebral discs in MR spine images is typically challenging for the following reasons: (1) object shapes are deformed and rotated; (2) the contrast between an object and its surroundings can be very low, which renders the boundary unclear; (3) the intensity within an object is not uniform.

Segmentation of intervertebral discs in MR spine images has been extensively studied. Ayed et al. [3] studied the application of graph-cut method for intervertebral disc segmentation and Michopoulou et al. [4] sought to detect and segment intervertebral discs using atlas-based and fuzzy clustering methods. Law et al. [5] proposed a detection and segmentation method for intervertebral discs using anisotropic oriented flux, while Rabia et al. [6] proposed a 3D intervertebral disc segmentation algorithm using a simplex active surface model using weak shape prior. However, performance of these conventional methods, which depend on mathematical algorithms with hand-crafted features, is limited by the challenges mentioned above.

Recent years have witnessed remarkable advances in the field of machine learning, especially with the use of deep-learning techniques. Convolutional neural networks (CNNs) effectively extract image features and perform effective classification based on these features. Several intelligent techniques, such as computer aided diagnoses that employ CNNs, have been reported in the field of medical imaging [7]. Ji et al. [8] attempted segmentation of intervertebral discs in MR spine images using a classification network by splitting the entire image into small patches.

**Figure 1.**
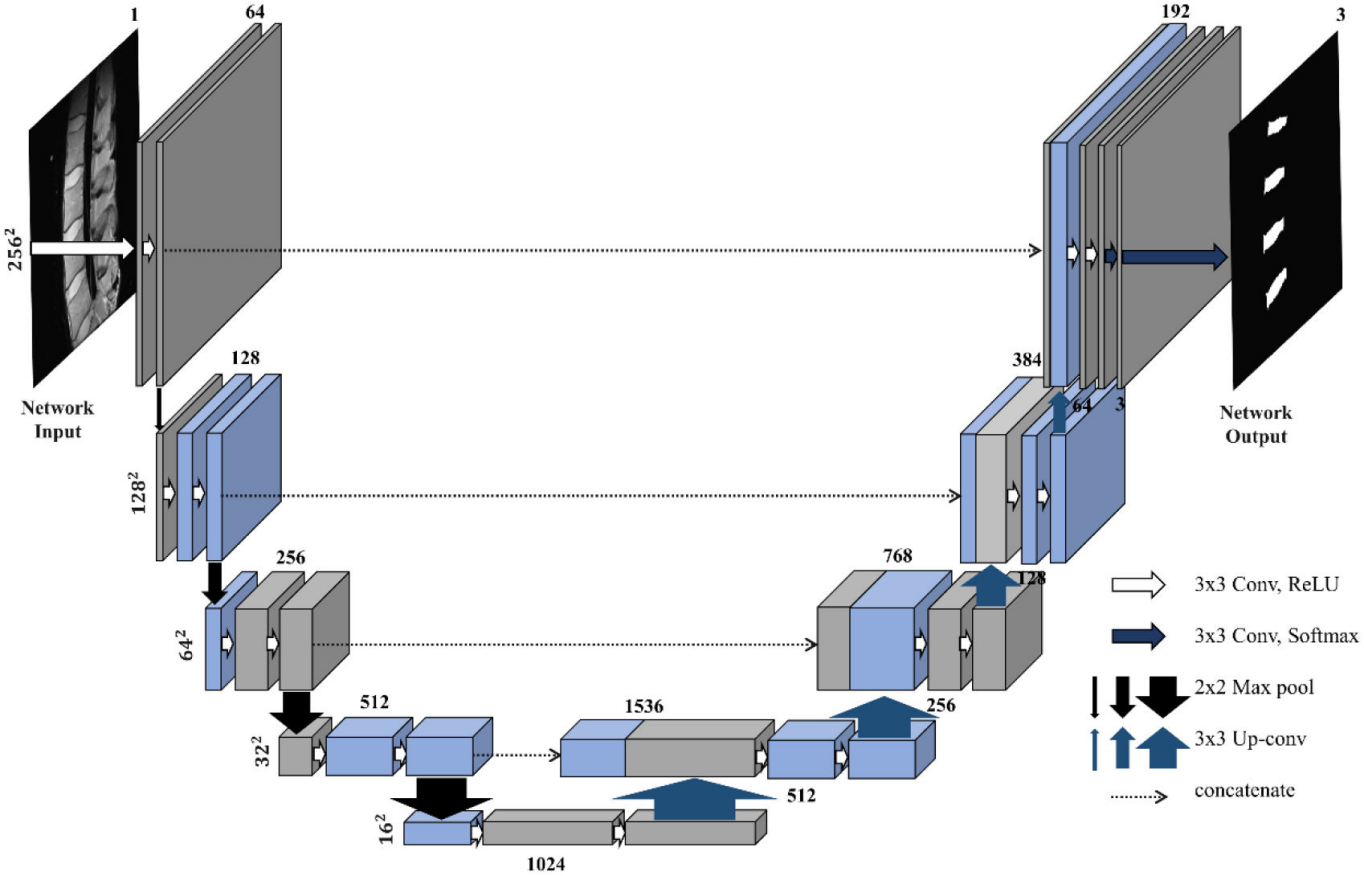
Structure of conventional U-network (U-net).

**Figure 2.**
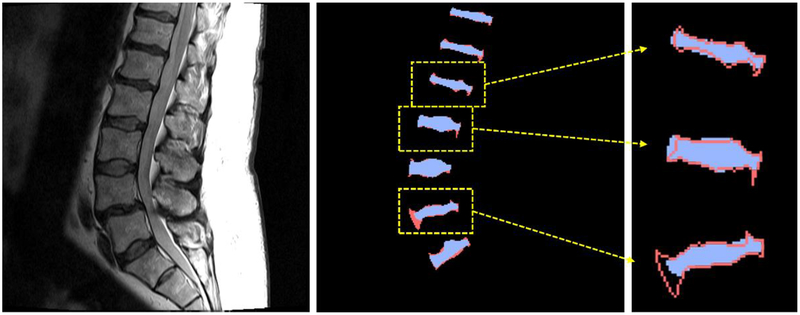
Intervertebral disc segmentation results from the conventional U-net. Blue areas are the results from the conventional U-net and red areas are manually segmented labels. Red lines are the boundaries of the labels.

The most common and effective CNN in medical image segmentation is the U-network (U-net) proposed by Ronneberger et al. [9]. As shown in Figure 1, a U-net is composed of an encoding part and a decoding part. The encoding part of conventional U-net is composed of convolutional layers and pooling layers and the decoding part is composed of convolutional layers and up-convolutional layers. Conventional U-net performs efficient feature extraction and segmentation using a large receptive field obtained through this structure [8]. However, since conventional U-net is based on feature extraction network for image classification, information pertaining to fine details of the image may disappear during the pooling process in the encoding part. For example, max-pooling layers, which is commonly used in U-nets, retains a pixel with the largest value among the neighboring four pixels and removes the information of the other pixels. Therefore, the pooling layer helps to efficiently detect the dominant information representing image characteristics, albeit with a loss of detailed information. The missing detail is not restored during up-convolutional layers. A skip connection can be added to this network to overcome this problem; however, it cannot completely recover the finer details. As a result, low-frequency information of the image is generally emphasized [10,11]. Figure 2 displays a comparison between the results of the conventional U-net segmentation and manually segmented labels. Dice similarity coefficient (DSC) [12] of segmentation for a whole area of intervertebral discs is 87.49%, while the DSC at the boundaries of the discs is as low as 40.87%. This suggests that it is difficult to achieve fine grain segmentation with conventional U-net and it may lead to unsatisfactory results for complex objects, such as intervertebral discs.

Dilated convolution is a way to overcome this limitation. Dilated convolution uses filters of various sizes with various rates. It allows users to control the resolution in the feature extraction process and to enlarge the field of view (FOV) without increasing parameter and cost [13,14].

In this paper, we propose a new network which can effectively perform fine grain segmentation for intervertebral discs. In our proposed network, pooling layers are modified to compensate for the aforementioned drawbacks. Convolutional layers and network structure are also improved to maximize the efficiency of the overall segmentation network. A preliminary study of this method was partially presented at the annual meeting of International Society for Magnetic Resonance in Medicine (ISMRM) in 2018 [15].

## 2. Materials and Methods

### 2.1. Network Design: Boundary Specific U-Network (BSU-Net)

**Figure 3.**
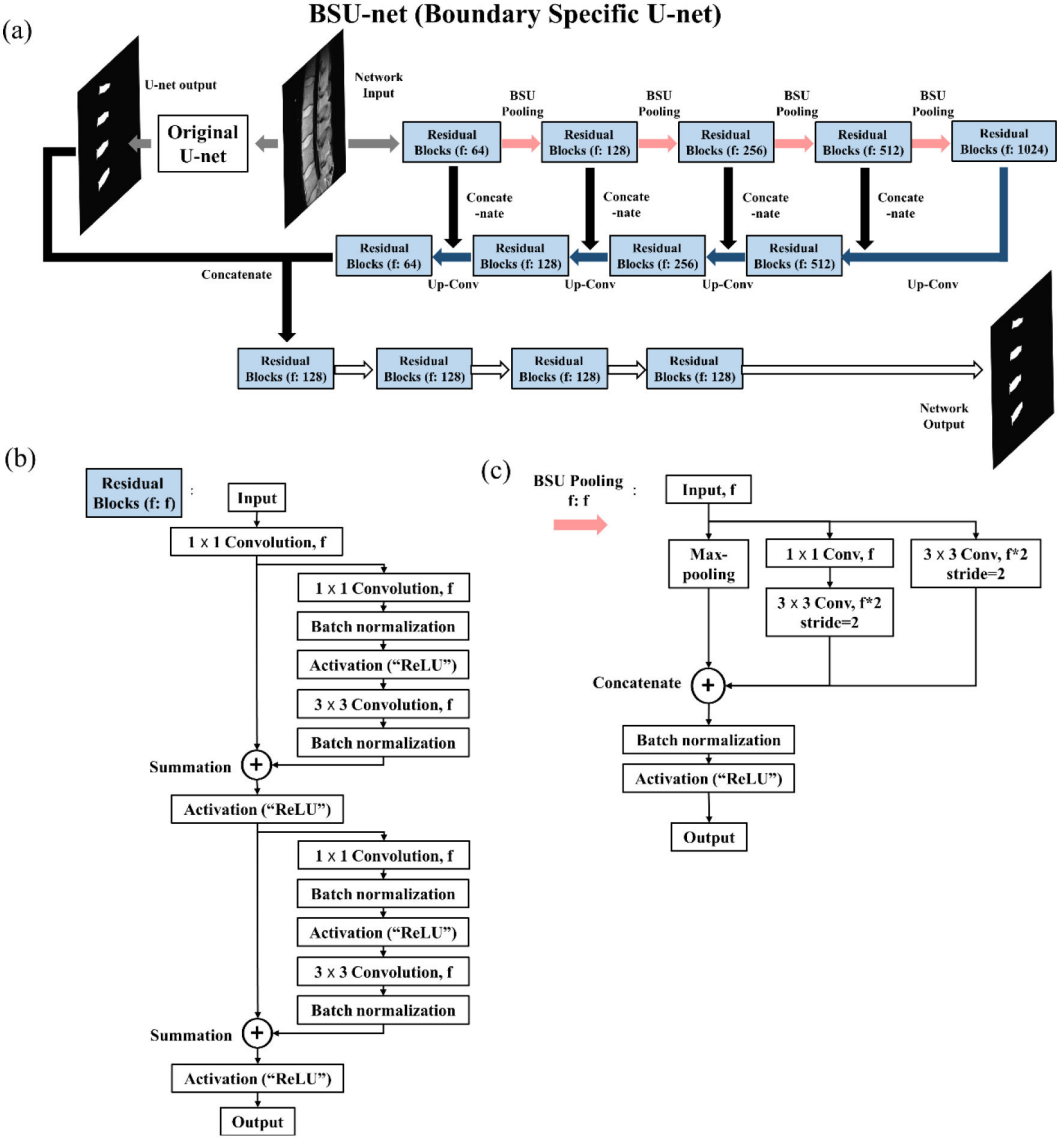
Whole structure of the proposed network. (**a**) Structure of the boundary specific U-network (BSU-net). (**b**) Structure of residual block. (**c**) Structure of BSU-pooling layer.

The purpose of this paper is to design a new network architecture based on U-nets, which can overcome the problems encountered in the detailed segmentation tasks. Hence, we propose a boundary specific U-network (BSU-net). The proposed network has a complex form of pooling layers and convolutional layers which are referred to as BSU-pooling layers and residual blocks respectively, and has a cascaded structure that uses preliminary outcomes of conventional U-net for efficient network learning. A schematic illustration of BSU-net is shown in Figure 3.

#### 2.1.1. BSU-Pooling Layer

BSU-net has three components. The first is the advanced pooling process. Conventional max-pooling layer used in conventional U-net discards rest of the pixels in a calculation field except for one pixel with maximum value. This process contributes to the efficiency of feature extraction; however, the loss of the information contained in the discarded pixels during the pooling process results in an inaccurate estimation of boundaries of target object in detailed segmentation tasks. Therefore, there is a need for an advanced pooling layer scheme that can minimize the loss of information while increasing the efficiency of feature extraction. The proposed BSU-pooling layer shown in Figure 3c uses both a max-pooling layer that increases the efficiency of feature extraction and convolutional layers that compute the neighboring information without discarding it. In this case, the stride of the convolutional layers is set to 2, so that down-sampling effect as in the max-pooling layer is possible. Furthermore, the inputs of the layer are preserved through multiple paths: a path passing through 3 × 3 convolutional layer and a path passing through 1 × 1 convolutional layer and another subsequent 3 × 3 convolutional layer (Figure 3c).

#### 2.1.2. Residual Block

**Figure 4.**
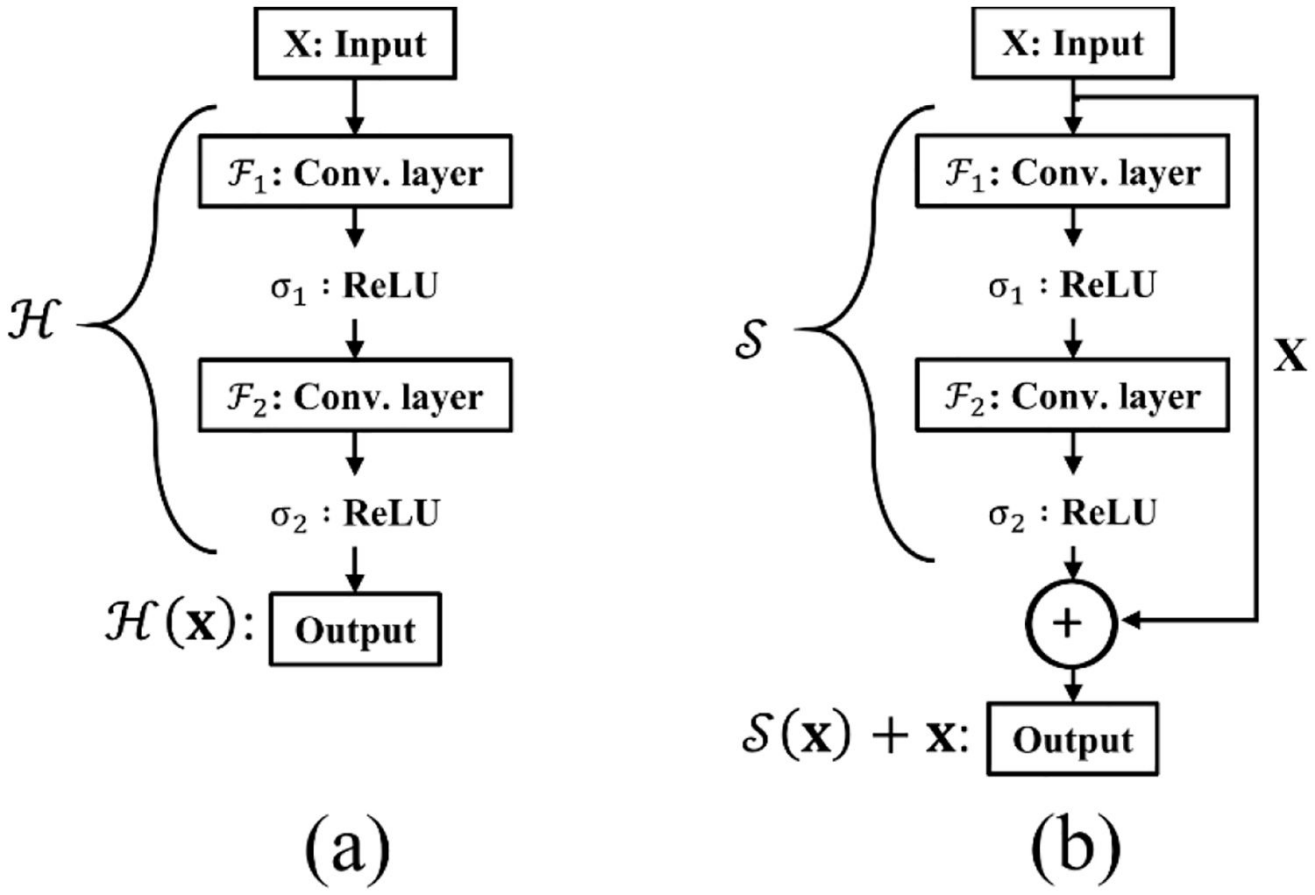
Introduction of residual learning. (**a**) Conventional neural network layers. (**b**) A learning network of residual function S.

The second component of BSU-net is the application of residual learning. Residual learning is applied to improve the efficiency of the convolutional layer. Conventional U-net is a very deep neural network with a large number of convolutional layers. Conventional U-net used in this study has a total of 38 convolutional layers and 62,803,650 learning parameters. Use of such a large number of consecutive convolutional layers can lead to the problem of gradient vanishing, which can degrade learning efficiency. The concept of residual learning was introduced to solve this problem [16]. Suppose we have a simple network H which is a part of a certain deep neural network. When H consists of two convolutional layers $F_{n}$ and $F_{n+1}$ and activation functions σ as shown in Figure 4a, output for the network with an input vector x is defined as $H\left(x\right)=\;\sigma_{n+1}\left(F_{n+1}\left(\sigma_{n}(F_{n}\left(x\right))\right)\right),\;x\in R^{w\times h\times c}$ where w, h, and c, respectively, denote the width, height, and the number of channels. During back propagation, gradient vanishing can occur if the weights of $F_{n}$ or $F_{n+1}$ are close to zero [16]. But if we change the network output $H\left(x\right)$ to $H\left(x\right)\;-\;x$, gradient vanishing is avoided. The changed network S is defined as $S\left(x\right)=\;H\left(x\right)\;–\;x$ and is also expressed as $H\left(x\right)=\;S\left(x\right)\;+\;x.\;H$ is converted to S with “shortcut connection” between input and output as shown in Figure 4b. In this case, gradient vanishing rarely occurs because 1 is added to $\frac{\partial S\left(x\right)}{\partial x}$. This change improves learning efficiency and allows the network to respond appropriately to small changes in input [16]. Residual block embeds this residual learning in BSU-net as displayed in Figure 3b. The first 1 × 1 convolutional layer immediately after the input is arranged to match filter size.

#### 2.1.3. Cascaded Network

Several studies have revealed that cascaded learning of networks improves learning efficiency and network performance [17–19]. It is an efficient way to improve performance of an entire network to provide outcomes from other networks or to combine outcomes from multiple networks like ensemble networks [20–22]. As shown in Figure 3a, conventional U-net outcomes are used to guide the learning of the entire BSU-net. This augments overall segmentation and fine grain segmentation and results in improved overall performance of the network.

### 2.2. Experimental Materials

The dataset used in the experiments comprised of 3D MR spine images of 20 patients sourced from Spineweb [23, 31]. Among this dataset, the images used in actual experiments are 1 to 3 mid-sagittal images per patient, totaling 25. The pixel size of images is 1.5 × 1.5 mm. Label data were made manually by a spine MR researcher and reviewed by a radiologist with an experience of more than 10 years. The experiments were implemented using 5-fold cross validation and each experiment had 5 test images and 20 training images. For fair validation of the network, all images from a single patient were used exclusively for either training or test.

The segmentation accuracy was evaluated using a DSC [12], and to assess the accuracy of measurement of fine details the evaluation was divided into the following three parts: (1) whole area; (2) boundary area; (3) boundary area with 2 pixels’ thickness. The first part evaluates segmentation accuracy of the entire area of intervertebral discs. The second and third parts evaluate the accuracy of the boundaries of the intervertebral discs whose boundary thickness was defined as 1 pixel and 2 pixels, respectively. A modified Hausdorff distance (MHD) was also used to evaluate the segmentation accuracy [24]. Smaller MHD indicates the better segmentation performance. Paired *t*-test [25] was used to compare the results for three types of measurements; *p*-values below 0.05 were considered statistically significant.

Conventional U-net and dilated U-net were compared with BSU-net. Dilated U-net is a network in which dilated convolution is applied to conventional U-net. In the structure of dilated U-net used in this study, max-pooling layers used in conventional U-net are replaced with convolutional layers with stride 2, and dilated convolution blocks are placed before each convolutional layer with stride 2. Dilated convolution blocks are composed of three concatenated dilated convolutional layers whose rate is 1, 2, and 3 respectively, and a convolutional layer placed after them. Activation function (rectified linear unit (ReLU)) and batch normalization were used after each convolutional or dilated convolutional layer.

The proposed network and all the neural networks used in our experiments were trained and tested using Google tensorflow library based on python 2.7 (Google, Mountain View, CA, USA) [32]. The computing hardware used in the experiments were as follows: GPU, NVIDIA GeForce GTX 1080 (NVIDIA Corp., Santa Clara, CA, USA); CPU, 3.60 GHz Octa core (Xeon, Intel, Santa Clara, CA, USA); Memory, 32 GB. Hyper parameters applied to the experiments were as follows: Learning rate was 10^−3^, total training epoch was 200, and optimizer was Adam. All images used as input for the networks were resized to 256 × 256 size matrix and normalized to values between 0 and 1.

## 3. Results

**Figure 5.**
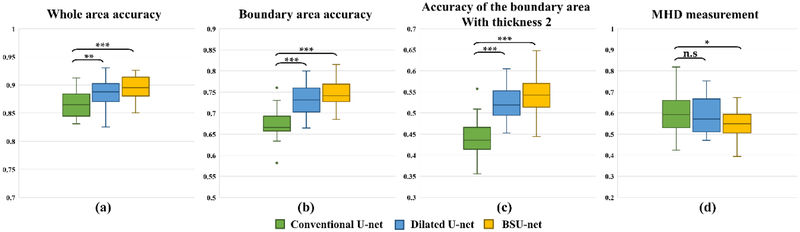
Segmentation results of networks. (a) Dice coefficients for whole area of intervertebral discs. (b) Dice coefficients of the boundaries of intervertebral discs whose thickness is defined as 1 pixel. (c) Dice coefficients of the boundaries of intervertebral discs whose thickness is defined as 2 pixels. (d) MHDs of intervertebral discs. A paired t-test was performed to calculate *p*-values. * denotes *p* < 0.05, ** denotes *p* < 0.01, *** denotes *p* < 0.001, and n.s. denotes not significant (*p* > 0.05).

**Figure 6.**
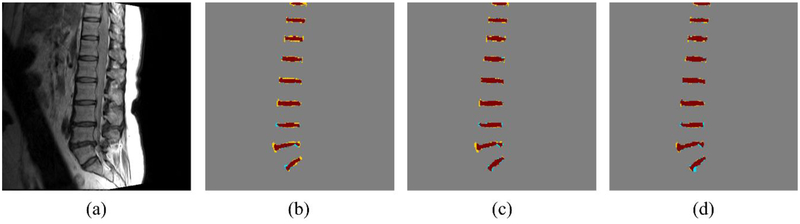
Segmentation result from networks. Brown area, yellow area, and blue area denote correctly segmented area, under-segmented area, and over segmented area, respectively. (**a**) Input image. (**b**) U-net result. (**c**) Dilated U-net result. (**d**) BSU-net result.

As shown in Table 1, both dilated U-net and BSU-net showed better results than conventional U-net in all DSC measurements. Furthermore, BSU-net showed better results than dilated U-net. As observed from these common trends, application of cascaded learning, BSU-pooling, and residual learning improved segmentation performance. In DSC measurement 1 (whole area segmentation), dilated U-net showed 2.02% higher DSC than conventional U-net and BSU-net showed a 3.00% higher DSC than conventional U-net. In DSC measurement 2 (boundary segmentation, thickness = 1 pixel), dilated U-net showed 8.29% higher DSC than conventional U-net and BSU-net showed 10.45% higher DSC than conventional U-net. In DSC measurement 3 (boundary segmentation, thickness = 2 pixels), dilated U-net showed 5.66% higher DSC than conventional U-net and BSU-net showed 7.34% higher DSC than conventional U-net. MHD results for three different networks showed similar trends (Table 2). Dilated U-net showed 0.03 mm lower MHD than conventional U-net and BSU-net showed 0.08 mm lower MHD than conventional U-net. Figure 5 compares the distributions of results according to the three DSC measurements and MHD measurement. In three DSC measurements, dilated U-net and BSU-net showed significant improvement in performance over conventional U-net. In DSC measurement 1, dilated U-net showed significantly increased DSC compared to conventional U-net (*p* < 0.01) and BSU-net showed significantly higher DSC compared to conventional U-net (*p* < 0.001). In DSC measurements 2 and 3, both dilated U-net and BSU-net showed significantly higher DSC than conventional U-net (*p* < 0.001) On the other hand, in MHD measurement, dilated U-net showed no statistical difference compared to conventional U-net (*p* > 0.05), while BSU-net showed statistically significant MHD compared to conventional U-net (*p* < 0.05). Figure 6 shows the comparisons between three networks. It is noticeable that under-segmented area in the boundaries of intervertebral discs decreased in order of Figure 6b–d and correctly segmented area increased in order of Figure 6b–d. This indicates that BSU-net segmented more accurately than the other two networks.

BSU-net has three components: BSU-pooling layer, residual block, and cascaded network. Table 3 shows the results of five different networks including U-net, BSU-net and three different networks applying several BSU-net components (BSU-pooling layer, BSU-pooling layer and residual block, and cascaded learning network). When pooling layers of U-net were replaced with BSU-pooling layers, the results of three DSC measurements and MHD measurement were improved compared to conventional U-net. The applications of residual blocks and BSU-pooling layers (i.e., BSU-layers) to U-net improved the results of all DSC measurements compared to conventional U-net while there was little increasement of MHD result. Cascaded U-net has a similar structure to BSU-net, but conventional convolutional layers and pooling layers are used instead of BSU-layers. Cascaded U-net showed higher DSC and smaller MHD compared to conventional U-net. The application of each component improved the segmentation performance in most cases.

**Figure 7.**
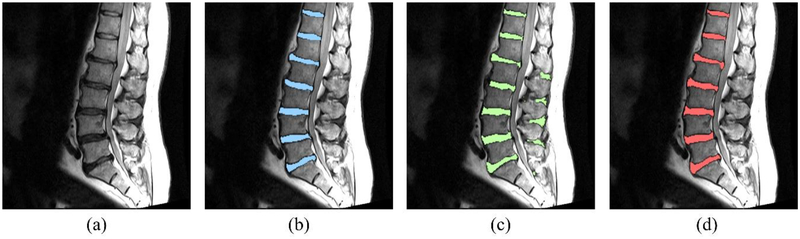
Segmentation results of the networks overlaid on the input image. (**a**) The input magnetic resonance (MR) image. (**b**) The input MR image with U-net segmentation result. (**c**) The input MR image with the result from the modified U-net which is the conventional U-net whose convolutional and pooling layers are replaced with BSU-layers. (**d**) The input MR image with BSU-net result.

**Figure 8.**
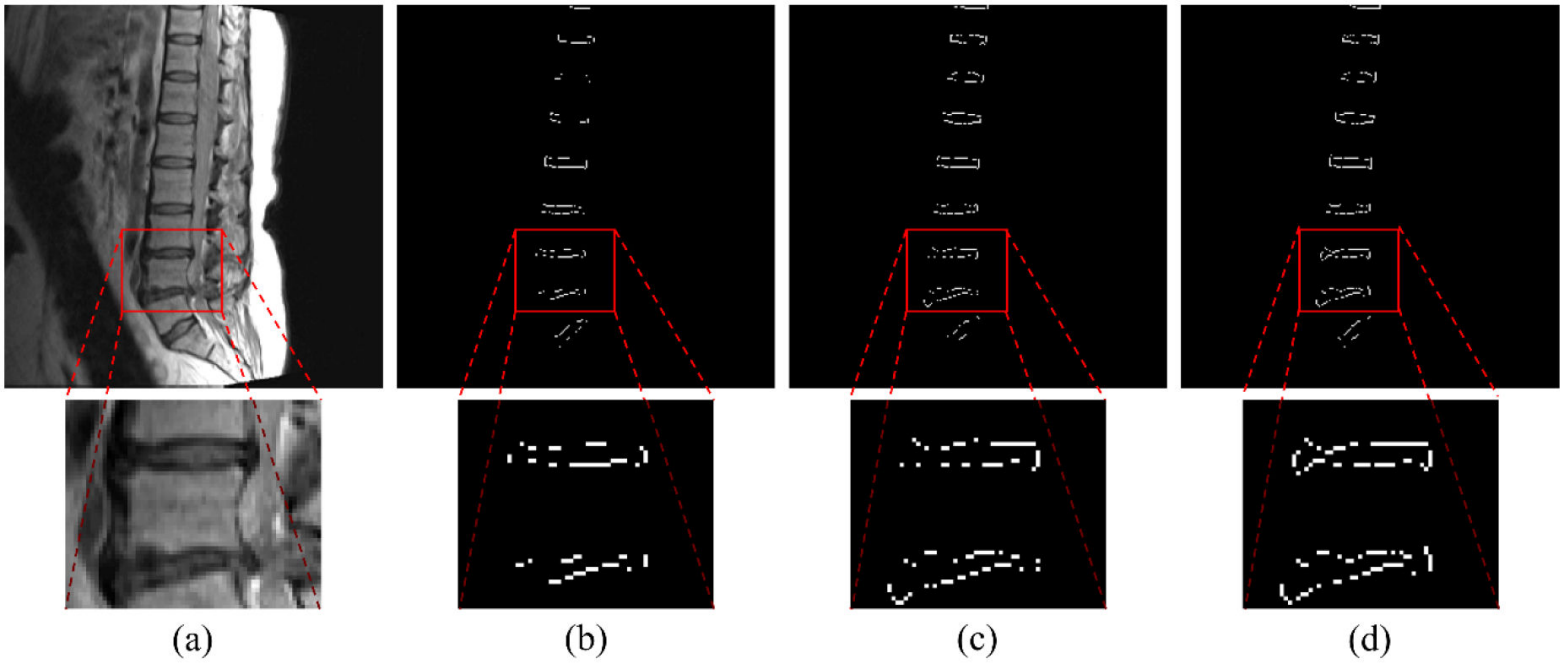
Segmentation results. (**a**) Input MR spine image. (**b**) Boundary segmentation result from U-net. (**c**) Boundary segmentation result from cascaded U-net. (**d**) Boundary segmentation result from BSU-net. White pixels correspond to boundary pixels that were perfectly matched with true boundary labels. BSU-net preserved more boundaries than other models.

**Figure 9.**
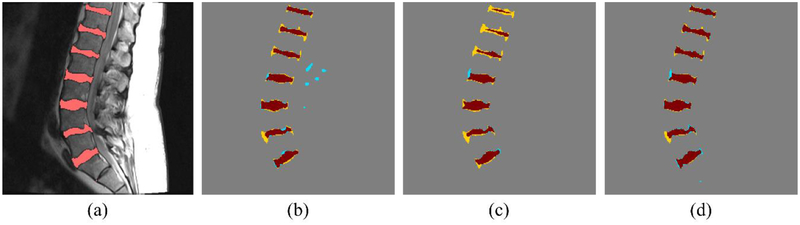
Segmentation results from all networks illustrating the outlier case of cascaded U-net. Brown area, yellow area, and blue area denote correctly segmented area, under-segmented area, and over segmented area, respectively. (**a**) Input image with label. (**b**) U-net result. (**c**) Cascaded U-net result. (**d**) BSU-net result.

Figures 7–9 show the results of the five different networks in Table 3. Figure 7b–d shows segmentation results of conventional U-net, U-net applying BSU-layers, and BSU-net, respectively. U-net applying BSU-layers segmented more delicately than conventional U-net, but there are some incorrectly segmented areas. On the other hand, the results of BSU-net have detailed boundaries and no incorrectly segmented area. Figure 8b–d shows segmentation results of conventional U-net, cascaded U-net, and BSU-net, respectively. The white pixels represent estimated boundary pixels that are perfectly matched with true boundary labels. It is easily noticeable that cascaded U-net found a higher number of true boundary pixels than conventional U-net, and BSU-net detected the most among the three different networks. The enlarged views at the bottom of Figure 8 clearly show the results from each and demonstrate the improved performance of BSU-net. Figure 9b–d also shows segmentation results of conventional U-net, cascaded U-net, and BSU-net, respectively. In this case, cascaded U-net did not properly segment intervertebral disc, and its results are worse than those of conventional U-net. In some cases of cascaded U-net, it segmented intervertebral discs smaller than their actual size. On the other hand, BSU-net showed successful performance in these cases. Standard deviations in Table 3 shows the stability of BSU-net. Standard deviations of BSU-net are the lowest in most accuracy measurements while those of cascaded U-net are the highest in most accuracy measurements.

## 4. Discussion

Conventional U-net is a commonly used deep learning network that displays good performance in various kinds of studies. It is used for segmentation of organs and cancers in various types of medical images [26–28], and it is also used for object segmentation of optical images [29]. However, conventional U-net has limited ability for detailed boundary segmentation [10] due to the structural limitations of a max-pooling layer that plays a key role in feature extraction process. It is not suitable for segmentation of objects with complex boundaries, such as intervertebral discs. The purpose of our proposed network, BSU-net, is to improve the pooling layer of conventional U-net. In this paper, BSU-net showed a better performance than conventional U-net for intervertebral disc segmentation in MR spine images. This indicates that BSU-net can perform more precise and fine-grain segmentation than conventional U-net. BSU-net will be of value in MR studies where quantitative MR values of disc need to be determined.

**Figure 10.**
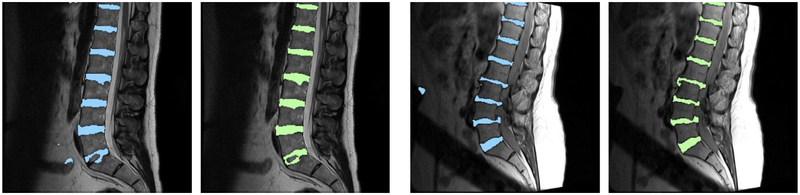
Comparison between dilated U-net and BSU-net. Blue area denotes segmentation results of dilated U-net and green area denotes segmentation results of BSU-net.

As shown in Tables 1 and 2 and Figure 5, dilated U-net performed better than conventional U-net and BSU-net showed better performance than dilated U-net. In most accuracy measurements, dilated U-net showed statistically significant performance improvement, but the improvement in MHD measurement was quite small. MHD indicates the accuracy of boundaries because it is based on the distances between obtained boundaries and reference boundaries. This indicates that the results of dilated U-net have many incorrectly segmented areas. Figure 10 shows the results of dilated U-net and BSU-net. There are some incorrectly segmented areas in the results of dilated U-net while the results of BSU-net have no incorrectly segmented areas. This is because the feature extraction process of dilated U-net did not remove unnecessary information compared to BSU-net. The number of trainable parameters used in BSU-net is 53,740,674 which is approximately 22% lower than dilated U-net (69,048,584) and approximately 14% lower than conventional U-net (62,803,650). This indicates that BSU-net performed successful fine-grain segmentation efficiently.

The components of the BSU-net are the BSU-pooling layer and residual block, and cascaded network. As shown in Table 3, the application of each component contributed to performance enhancement. The performance improvement of applying residual blocks is much smaller than those of applying other components. However, the number of trainable parameters were approximately 12% decreased. Therefore, the application of residual blocks brought efficiency to the entire learning.

When BSU-layers were applied to U-net, the result of DSC measurement 1 was only 0.74% higher than conventional U-net. The application of BSU-layers brought improved performance in terms of fine-grain segmentation, given the fact that the result of DSC measurement 2 was 7.72% higher than conventional U-net and the result of accuracy measurement 3 was 4.18% higher than conventional U-net. However, the MHD result of U-net applying BSU-layers is worse than conventional U-net. These results indicate that the results of U-net applying BSU-layers had many incorrectly segmented areas. Figure 7 shows many incorrectly segmented areas in the results of U-net applying BSU-layers and they decreased the accuracy of whole segmented areas. These incorrectly segmented areas occurred because BSU-layers preserved the detailed information which was discarded in the feature extraction process in conventional U-net. The retention of this information affected the performance of the network. Therefore, in order to fully utilize the advantages of BSU-layers, there is a need for a guiding mechanism that can discard unnecessary parts and narrow the target area into proper regions. Cascaded learning method can use the outcomes of conventional U-net to effectively guide BSU-layers to focus on the proper regions. This is the reason why BSU-net, which combines cascaded learning method and BSU-layers at the same time, can achieve a high performance. Figure 7d shows the successful segmentation results of BSU-net without incorrectly segmented area. Appropriate guidance for BSU-layers improved the efficiency of the entire network.

In general, cascaded learning uses the outcomes of former networks as inputs at the beginning of following networks [17–19]. However, cascaded learning applied to BSU-net puts the outcomes of conventional U-net at the back-end rather than the beginning of the following network. This is because detailed information of conventional U-net outcomes disappeared during the pooling process in the encoding part of the network. A network showed 1.67%, 4.01%, and 2.92% lower accuracy for three DSC measurements respectively when the outcomes of conventional U-net were put into the initial part of the following network.

As shown in Table 3, standard deviations of cascaded U-net are highest in most accuracy measurements. Figure 9 also shows the unstable performance of cascaded U-net. For eight out of the 25 cases, cascaded U-net showed over 1% lower accuracy than conventional U-net in all eight cases; two of these showed more than 7% lower accuracy. Contrastingly, BSU-net showed lower accuracy than conventional U-net in just one case where the difference is smaller than 1%. This is because important information pertaining to the boundary areas was discarded during the feature extraction process in cascaded U-net. The loss of important information in the max-pooling process is a noticeable problem. On the other hand, BSU-net distinguished most intervertebral disc areas correctly, while unsegmented areas and over-segmented areas did not deviate much from the actual boundaries. These results also indicate that the application of BSU-layers to cascaded U-net provides stability and generality to the network. Furthermore, the use of BSU-layers enables efficient training of the network. Cascaded U-net used in our experiments has 63,912,898 trainable parameters in a total of 42 convolutional layers (3 × 3 convolutional layers: 41 and 1 × 1 convolutional layer: 1), while BSU-net has 53,740,674 trainable parameters, approximately 16% less than that in cascaded U-net, in a total of 79 convolutional layers (3 × 3 convolutional layers: 35 and 1 × 1 convolutional layer: 44).

## 5. Conclusions

Intervertebral disc segmentation in MR images is challenging owing to their complex shapes and non-uniform intensity. This study introduces a robust deep-learning segmentation network, boundary specific U-net (BSU-net), which can successfully segment intervertebral discs with complex boundaries.

Conventional U-net is a deep learning segmentation algorithm for image segmentation which is commonly used in various fields. However, conventional U-net is not suitable for intervertebral disc segmentation because its performance with respect to the details of segmentation (such as the boundaries) is still limited owing to the structural limitations of the max-pooling layer that plays a key role in the feature extraction process in conventional U-net. The proposed BSU-net can overcome the limitations of conventional U-net and achieve fine-grain segmentation. BSU-net uses modified convolutional and pooling layers and applies cascaded learning method to overcome the structural limitations of conventional U-net. BSU-net performed intervertebral discs segmentation in MR spine images with higher accuracy than conventional U-net, especially in the boundary areas.

Obtaining specific information about intervertebral discs is of great help for the diagnosis and treatment of lumbar diseases. In many translational studies with real patients, quantitative MRI such as T_2_ mapping is used to show treatment efficiency or track subtle changes over time. BSU-net, though not clinically applicable at this time, will be of great value in translational MR studies where quantitative MR values of the disc need to be determined using regions of interest. Our finding of 89% Dice similarity coefficient of BSU-net against human annotator compares favorably with inter-observer agreement of about 80% [30].

## Figures and Tables

**Table 1.** Dice similarity coefficient (DSC) measurements for the three different models. Accuracy for boundary area is very limited.

		Mean (%)	SD (%)
Whole area segmentation	U-net	86.44	2.24
	Dilated U-net	88.46	2.63
	BSU-net	89.44	2.14
Boundary segmentation (thickness = 1 pixel)	U-net	44.16	4.18
	Dilated U-net	52.45	4.08
	BSU-net	54.62	4.59
Boundary segmentation (thickness = 2 pixels)	U-net	67.51	3.59
	Dilated U-net	73.17	3.70
	BSU-net	74.85	3.20

**Table 2.** Modified Hausdorff distance (MHD) measurements for the three different models.

	Mean (mm)	SD (mm)
U-net	0.89	0.14
Dilated U-net	0.86	0.14
BSU-net	0.81	0.10

**Table 3.** DSC and MHD measurements for five different networks including conventional U-net, BSU-net and three different networks applying several components of BSU-net.

	DSC (%)			MHD (mm)
	Measurement 1	Measurement 2	Measurement 3	
Conventional U-net	86.44±2.24	44.16±4.18	67.51±3.59	0.89±0.14
U-net + BSU-pooling layer	87.30±3.16	50.68±5.50	71.68±4.76	0.88±0.14
U-net + BSU-layer	87.19±2.67	51.88±5.67	71.68±5.48	0.90±0.18
Cascaded U-net	87.70±4.00	50.25±8.68	71.33±7.63	0.86±0.17
BSU-net	89.44±2.14	54.62±4.59	74.85±3.20	0.81±0.10
